# NF-κB p65 repression by the sesquiterpene lactone, Helenalin, contributes to the induction of autophagy cell death

**DOI:** 10.1186/1472-6882-12-93

**Published:** 2012-07-11

**Authors:** Chuan Bian Lim, Pan You Fu, Nung Ky, Hong Shuang Zhu, XiaoLing Feng, Jinming Li, Kandhadayar Gopalan Srinivasan, Mohamed Sabry Hamza, Yan Zhao

**Affiliations:** 1 Division of Chemical Biology and Biotechnology, School of Biological Sciences, College of Science, Nanyang Technological University, 60 Nanyang Drive, Singapore 637551, Singapore; 2 HeiLongJiang University of Chinese Medicine, Harbin, People’s Republic of China; 3 National Cancer Centre of Singapore, NCCS-VARI Translational Research Laboratory, #501, Level 5, 11 Hospital Drive, Singapore 169610, Singapore; 4 1stBASE Pte Ltd., 41 Singapore Science Park II, The Gemini, Singapore 117610, Singapore; 5 MSD, Translational Medicine Research Center, 8 Biomedical Grove, #04-01/-05 & #05-01/05, Neuros Building, Singapore 138665, Singapore

**Keywords:** Helenalin or Hele(Helenalin), Autophagy, Caspase, NF-κB, Atg12 and LC3-B

## Abstract

**Background:**

Numerous studies have demonstrated that autophagy plays a vital role in maintaining cellular homeostasis. Interestingly, several anticancer agents were found to exert their anticancer effects by triggering autophagy. Emerging data suggest that autophagy represents a novel mechanism that can be exploited for therapeutic benefit. Pharmacologically active natural compounds such as those from marine, terrestrial plants and animals represent a promising resource for novel anticancer drugs. There are several prominent examples from the past proving the success of natural products and derivatives exhibiting anticancer activity. Helenalin, a sesquiterpene lactone has been demonstrated to have potent anti-inflammatory and antitumor activity. Albeit previous studies demonstrating helenalin’s multi modal action on cellular proliferative and apoptosis, the mechanisms underlying its action are largely unexplained.

**Methods:**

To deduce the mechanistic action of helenalin, cancer cells were treated with the drug at various concentrations and time intervals. Using western blot, FACS analysis, overexpression and knockdown studies, cellular signaling pathways were interrogated focusing on apoptosis and autophagy markers.

**Results:**

We show here that helenalin induces sub-G1 arrest, apoptosis, caspase cleavage and increases the levels of the autophagic markers. Suppression of caspase cleavage by the pan caspase inhibitor, Z-VAD-fmk, suppressed induction of LC3-B and Atg12 and reduced autophagic cell death, indicating caspase activity was essential for autophagic cell death induced by helenalin. Additionally, helenalin suppressed NF-κB p65 expression in a dose and time dependent manner. Exogenous overexpression of p65 was accompanied by reduced levels of cell death whereas siRNA mediated suppression led to augmented levels of caspase cleavage, autophagic cell death markers and increased cell death.

**Conclusions:**

Taken together, these results show that helenalin mediated autophagic cell death entails inhibition of NF-κB p65, thus providing a promising approach for the treatment of cancers with aberrant activation of the NF-κB pathway.

## Background

The efforts of many researchers during the past dozen years to identify novel compounds with anticancer activity have pointed to plants and herbs used in herbal medicine. The rationale behind this approach is that herbal medicine looks back on a 5000-years tradition. Hence, it can be expected that many medicinal herbs and plants have been selected for pharmacological activity [1]. Many studies have shown that herbal medicine is indeed a valuable resource for novel compounds with activity against tumors *in vitro* and *in vivo*[2-4]. Hence, the chances to find novel compounds with activity against tumor cells in natural product libraries are higher than in synthetic libraries.

In this regard, helenalin, a naturally occurring sesquiterpene lactone has generally been considered as a distinctly promising and potent antitumor compound. Helenalin has been shown to be a potent inhibitor of hTERT (human Telomerase Reverse Transcriptase) and telomerase in hematopoietic cancer cell [5], induces apoptosis in activated CD4+ T cells through the mitochondrial apoptosis pathway [6] and have been shown to selectively alkylate the p65 subunit of NF-κB [7].

In this report, we provide a mechanism by which NF-κB p65 plays a significant role in modulating autophagy induced cell death by the sesquiterpene lactone, helenalin. NF-κB p65 expression is down regulated upon helenalin treatment in a time and dose dependent manner. Down regulation of NF-κB p65 in turn induces caspase cleavage and autophagic genes Atg12 and LC3-B resulting in sub-G1 arrest and cell death. Exogenous expression of NF-κB p65 attenuates caspase cleavage and subsequently autophagy, demonstrating a mechanistic pathway of helenalin induced autophagic cell death. siRNA mediated transcriptional knockdown of NF-κB p65, Atg12 or LC3-B or inhibition of caspase cleavage using Z-VAD-fmk diminishes autophage cell death. In addition, helenalin induced apoptosis by activating the intrinsic apoptosis pathway. Taken together, we surmise that helenalin mediated apoptotic and autophagic cell death may provide a promising treatment strategy for cancers with aberrant activation of the NF-kB pathway.

## Methods

### Cell Culture and drug treatment

A2780 (human ovarian cancer cell line), RKO (colon carcinoma cancer cell line) and MCF-7 (breast adenocarcinoma cancer cell line) were obtained from ATCC (Manassas, VA). Cells were cultured in Dulbecco’s modified Eagle’s medium, supplemented with 10 % fetal bovine serum, 1 % penicillin-streptomycin (all from GIBCO® Invitrogen, Carlsbad, CA) in a humidified 5 % CO2 atm at 37 °C. Cells were treated with helenalin (Ambrosa-2,11(13)-dien-12-oic acid, 6-α,8-β-dihydroxy-4-oxo-, 12,8-lactone) purchased from EMD biosciences (Gibbstown, NJ). Dimethyl sulfoxide was used throughout the experiments as the vehicle control. At least three biological experiments were performed to verify observations.

### Flow cytometry analysis

Cells were harvested after drug treatment and fixed with 70 % ethanol. Fixed cells were treated with RNase (100 μg/ml) and stained with propidium iodide (50 μg/ml). Subsequently, stained cells were analyzed for DNA content by flow cytometry using FACScalibur (Becton Dickinson, Franklin Lakes, NJ). Cell cycle fractions were quantifies using the CellQuest software (BD Biosciences, San Jose, CA). Further details can be found in [8]. At least three biological experiments were performed to verify observations.

### Cell Proliferation Assay

Inhibition of cell proliferation by helenalin was assessed using the MTT assay (Roche, Indianapolis, IN). Briefly, A2780, MCF-7 or RKO cells were plated in 96-well culture plates (5 × 10^4^ cells/well) and treated the following day with helenalin or DMSO vehicle as described in the results section. Following helenalin treatment, cells were incubated with MTT labeling reagent for 4 h, solubilized in 10 % SDS, and the MTT metabolite formazan crystals were quantitated at 575 nm on a microplate reader (Tecan, Männedorf, Switzerland). All experiments were performed and verified using at least three biological replicates.

### Clonogenic Assay

To determine the growth suppression effect of helenalin treatment, A2780 cells were treated with helenalin or DMSO vehicle for 24 h. After treatment, cells were replated in complete DMEM and allowed to grow for 14 days to form colonies that were then stained with crystal violet (Sigma) and quantified. All experiments were performed and verified using at least three biological replicates.

### Annexin-V measurements

Direct fluorescence staining of apoptotic cells for flow cytometric analysis was performed with the Annexin V-FITC apoptosis detection kit (BD Pharmingen, San Jose, CA). After the indicated times, cells were harvested and stained according to the manufacturer’s protocol. Stained cells were analyzed in a flow cytometer. All experiments were performed and verified using at least three biological replicates.

### Western Blotting

Western blotting procedure was followed according to [8]. Briefly, **c**ells were lysed in appropriate volume of lysis buffer (Sigma Aldrich, St. Louis, MO). 50 μg of protein samples were separated by SDS-PAGE and transferred onto nitrocellulose membrane (Bio-Rad, Hercules, CA). The membranes were immunoblotted with primary antibodies purchased from Cell Signal Technology (Danvers, MA) or Santa Cruz Biotechnology, Inc (Santa Cruz, CA). Blots were incubated with horseradish peroxide-conjugated goat anti-rabbit, goat anti mouse or rabbit anti-goat secondary antibodies purchased from Santa Cruz Biotechnology, Santa Cruz, CA. All experiments were performed and verified using at least three biological replicates.

### siRNA transfection

A2780 cells were transfected with non-targeting control siRNA (siRNA Neg), siRNA Atg12, siRNA LC3-B or siRNA RelA p65 when cells reached 80 % confluency. After 24 h, cells were split 1:3, and treated with helenalin or DMSO the next day. Final siRNA concentration was 100nM and transfection was performed using Lipofectamine RNAimax (Invitrogen, Carlsbad, CA) according to the manufacturer’s protocol. Target sequences used for siRNA against *Atg12*, *LC3-B* and RelA p65 were 5′CUUAACAGAUGUGAUCUAU-3′, 5′-GUAAUUCCAGCAGUAAUUU-3′, 5′-CUCAAGAUCUGCCGAGUGA-3′ respectively. All experiments were performed and verified using at least three biological replicates.

### Plasmid transfection

A2780 cells were transfected with 2.0 ug of empty vector or NF-κB RelA p65 overexpressing vector (purchased from Origene Technologies, Rockville, MD; Cat # RC220780) using FuGENE 6 transfection reagent (Roche, Indianapolis, IN) following manufacturer’s instructions. All experiments were performed and verified using at least three biological replicates.

### Acridine Orange staining for autophagy detection

Cell staining with Acridine orange (10 mg/ml in water, A8097, Sigma Chemical Co) was performed according to published procedures [9], adding at a final concentration of 1 mg/ml for a period of 15 min. Bafilomycin A1 (Sigma Chemical Co.) was dissolved in DMSO and added to the cells 45 min before addition of acridine orange. Photographs were obtained with a fluorescence microscope and percent of staining was determined by harvesting cells by trypsinization and measuring with a FACSCalibur from (Becton Dickinson) using CellQuest software. All experiments were performed and verified using at least three biological replicates.

## Results and Discussion

### Helenalin Inhibits Cell Proliferation and Clonogenic Survival in cancer cells

**Figure 1:**
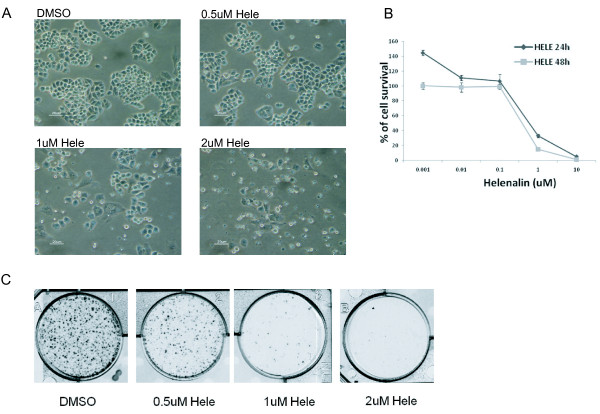
**Helenalin induces cytotoxicity in A2780 human ovarian cancer cell line Phase contrast micrographs of A2780 cells treated with increasing concentrations of helenalin of 0.5, 1.0 and 2uM for 24 h.** Changes in cell number and morphology were observed when compared to DMSO control. (**B**) Percent of viable cells after treatment with increasing concentrations of helenalin (serial dilution for drug concentration ranging from 10uM to 0.001uM) using the MTT assay. (**C**) Clonogenic survival assay. A2780 cells were treated with increasing concentrations of helenalin and then incubated for 2 weeks. The cells were stained with crystal violet for 16 h. Representative dishes are shown. All results represent data from three independent experiments.

To examine the effect of helenalin on cell proliferation and clonogenic survival, human ovarian cancer A2780 cells were treated with helenalin and effects on cell proliferation and survival was determined using phase contrast microscopy, crystal violet staining and MTT assays. Phase contrast micrographs of A2780 cells treated with increasing concentrations of helenalin of 0.5, 1.0 and 2uM for 24 h showed changes in cell number and morphology. Morphologic signs of apoptosis included changes such as membrane blebbing and apoptotic body formation (Figure 1A). To measure the amount of cell survival after helenalin exposure, A2780 cells were cultured in the presence of increasing doses of helenalin (serial dilution for drug concentration ranging from 10uM to 0.001uM) for 24 h and cell survival was determined using the MTT assay. As shown in Figure 1B, increasing concentrations of helenalin reduced the percentage of cell survival in a dose dependent manner. To further investigate the growth suppression effect of helenalin, we performed *in vitro* clonogenic assays. Figure 1C shows the effects of helenalin on the clonogenic potential of the control (DMSO) and helenalin-treated A2780 cells. Helenalin reduced clonogenicity of A2780 cells in a dose-dependent manner, where 2uM of helenalin completely suppressed clonogenic growth.

### Induction of G1 Phase Arrest and cell death by Helenalin

**Figure 2:**
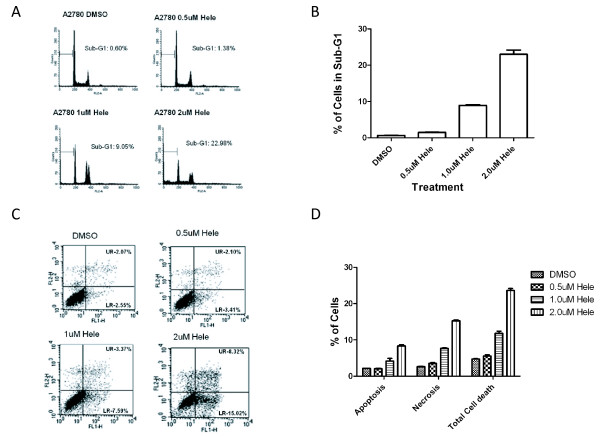
**Helenalin induces cell cycle arrest in G1 phase and modulates apoptosis in a dose-dependent manner.** (**A**) 1.85 × 105 cells/ml were seeded onto 6-well plates and incubated for 24 h. Various concentrations of helenalin were added to the culture medium and incubated for an additional 24 h. Cells were then harvested and analyzed by flow cytometry. The cell cycle phase distribution was determined using CellQuest software. (**B**) Percent of cells in Sub-G1 after drug treatment representing three independent experiments. (**C**) Measurement of apoptotic cell death by staining with FITC-Annexin V and Propidium Iodide. Lower right hand quadrant for each dose treatment represents percent of apoptosis, while upper right hand quadrant represents early necrotic cells. (**D**) Percent of cells that are either apoptotic, necrotic or total cell death as measured by staining cells with FITC-Annexin V and Propidium Iodide. Percentages are the average of three independent biological replicates.

**Figure 3:**
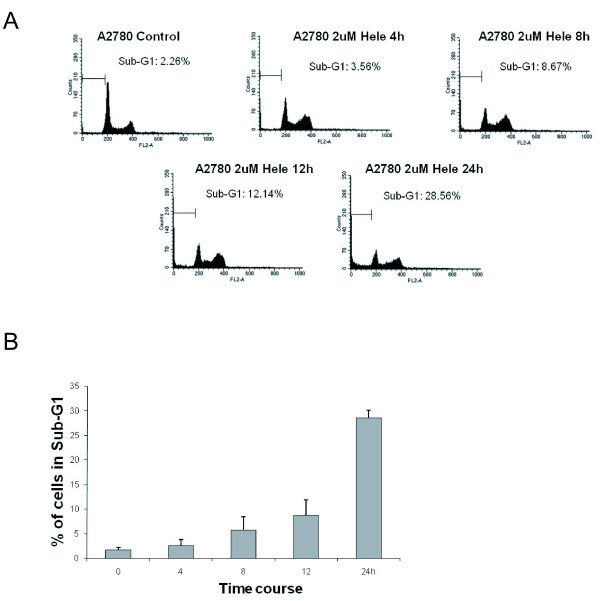
**Helenalin induces cell cycle arrest in G1 phase and modulates apoptosis in a time-dependent manner.** (**A**) 1.85 × 105 cells/ml were seeded onto 6-well plates and incubated for 24 h. 2uM of helenalin was added to the culture medium and incubated for 4,8,12 and 24 h. Cells were harvested and analyzed by flow cytometry. The cell cycle phase distribution was determined using CellQuest software. Figures represent data from three independent experiments. (**B**) Percent of cells in Sub-G1 after the indicated timepoints representing three biological experiments.

To identify the mechanism of helenalin-induced cell proliferation inhibition and cell death, we examined the effects of helenalin on cell cycle distribution by flow cytometry. As shown in Figure 2A, cells in sub-G1 phase increased in a helenalin dose dependent manner, analogous with the earlier observation that helenalin inhibited cell proliferation (Figure 1A). As much as 25 % of cells in sub-G1 were observed in A2780 cells treated with 2uM helenalin (Figure 2B). To rule out that our findings were cell line specific, we replicated our experiments in MCF-7 breast adenocarcinoma cancer cell line and RKO colon carcinoma cancer cell lines. As shown in Additional file 1: Figure S1 A and C, increase in sub-G1 levels were observed in these additional cancer cell lines, with the RKO cell line exhibiting greater sensitivity to helenalin whilst MCF-7 cells were comparatively less sensitive. As demonstrated previously for the A2780 cancer cell line (Figure 1B), helenalin also reduced the percentage of cell survival in a dose dependent manner in both the MCF-7 and RKO cancer cell lines (Additional file 1: Figure S1 B and D). As sub-G1 levels are an indicative measurement of cell death [10,11], we examined whether the fraction of cells in sub-G1 after helenalin treatment was attributable to cells undergoing apoptosis. We measured apoptotic cell death by staining with FITC-Annexin V and Propidium Iodide and performed flow cytometry to analyze sub fractions of cells undergoing apoptosis or necrosis. As revealed in Figure 2C, partial increase in apoptotic cells was observed after helenalin treatment when compared to the FACS data (Figure 2A,B), suggesting cells in sub-G1 undergo cell death *via* apoptosis in addition to other cell death mechanisms. In addition to the dose dependent effects of helenalin observed, we performed additional experiments to investigate the effects on A2780 cells exposed to helenalin at varying treatment times. Flow cytometry assays performed on cells harvested after different exposure times demonstrate an increase in sub-G1 levels with increasing exposure to helenalin (Figure 3A). As much as 35 % of cells are in sub-G1 24 h post treatment with helenalin (Figure 3B).

### Helenalin induces cell death *via* caspase cleavage and induction of autophagy

**Figure 4:**
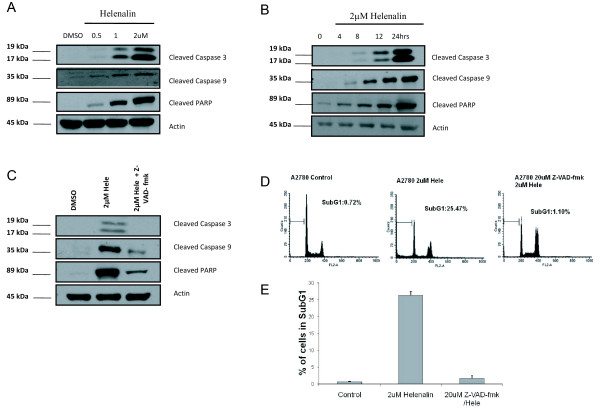
**Helenalin activates caspase cleavage.** A2750 cells were treated with increasing concentrations of helenalin for 24 h (**A**) or with 2uM helenalin for the indicated timepoints (**B**), following which cells were harvested and cell lysates prepared and subjected to immunoblot analysis for cleaved PARP, caspase 3 and 9. Actin was used as a loading control.A2750 cells were subjected to caspase inhibitor, Z-VAD-fmk treatment two hours before addition of 2uM helenalin for 24 h, following which the cells were lysed and subjected to (**C**) immunoblot analysis for cleaved caspases and PARP or (**D**) harvested and analyzed for cell cycle flow distribution using FACS analysis. (**E**) Percent of cells in Sub-G1 in DMSO, helenalin or Z-VAD-fmk plus helenalin treatment. All figures represent data from three independent experiments.

**Figure 5:**
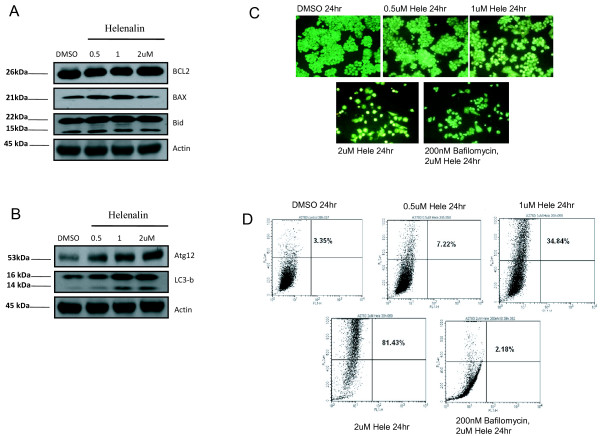
**Helenalin induces autophagy cell death.** A2750 cells were treated with increasing concentrations of helenalin for 24 h, following which cells were harvested and cell lysates prepared and subjected to immunoblot analysis for (**A**) BCL2, BAX and Bid or (**B**) Atg12 and LC3-B. Actin was used as the loading control. A2750 cells were treated with DMSO, 0.5uM, 1uM, 2uM helenalin or pretreated with 200nM bafilomycin A1 for 45 min before treatment with 2uM helenalin for 24 h. Following which, cells were stained with Acridine Orange Solution and photographed using a florescence microscope (positive AVO staining in red) (**C**) or (**D**) trypsinized and harvested for FACS analysis to quantitate levels of cell staining representing autophagy. Figures represent data from three independent experiments.

To further investigate the mechanistic action of cell death induced by helenalin, we performed western blot analysis to detect proteins that have been shown to be involved in both the intrinsic and extrinsic apoptosis pathways. Cells treated with increasing concentrations of helenalin were lysed and subjected to western blot analysis for cleaved caspases 3 and 9 and also for cleaved PARP. After 24 h of treatment, the levels of cleaved caspases increased with increasing concentrations of helenalin (Figure 4A). Using a dose of 2uM helenalin, it was observed that levels of cleaved caspase 3, 9 and PARP were detected at the outset of 8 h post treatment with subsequent increase in cleavage with protracted treatment times (Figure 4B). To substantiate the prerequisite of caspase cleavage as a determinant for helenalin induced cell death, we employed the use of the pan caspase inhibitor, Z-VAD-fmk to block caspase cleavage during helenalin treatment and determined the levels of sub-G1cells by flow cytometry. Addition of Z-VAD-fmk to cells prior to helenalin treatment suppressed caspase 3, 9 and PARP cleavage (Figure 4C) and levels of sub-G1 cells measured by flow cytometry showed comparable levels to those of control treated cells *versus* to those of cells treated with helenalin alone (Figure 4D). Quantitative measurements of cells in sub-G1 were reduced from levels of ~25 % in helenalin alone treated cells to less than 2 % with helenalin in combination with Z-VAD-fmk (Figure 4E). We subsequently investigated the intrinsic cell death pathway by assessing the protein levels of Bcl-2, Bax and Bid in lysates from cells treated with different concentrations of helenalin. As shown in Figure 5A, no appreciable differences in protein expression were observed suggesting that helenalin induced cell death was not attributable to activation of the Bcl-2, Bax and Bid. Interestingly, as shown in Figure 4, helenalin activated caspase 9, strongly suggesting helenalin induces intrinsic apoptotic cell death. We next investigated the levels of Atg12 and LC3-B, both biomarkers indicative of autophagy cell death. As demonstrated in Figure 5B, there was a dose dependent increase in protein levels of Atg12 and LC3-B with increasing concentrations of helenalin. Our findings are in contrast to recently published data where there was no increase of LC3-B detected in Jurkat cells treated with helenalin [12]. The authors of this manuscript can only speculate to the differences in observations as cell line specific. To verify that cells were indeed undergoing autophagy, cells were treated with varying concentrations of helenalin and stained with Acridine Orange solution to detect and measure acidic vesicular organelle (AVO) formation. As shown in Figure 5C, vital staining of cells with acridine orange showed the accumulation of AVO in the cytoplasm of cells exposed to increasing concentrations of helenalin. This was inhibited by addition of bafilomycin A1 (200 nM), an H^+^ATPase inhibitor (Figure 5C). The amount of AVO staining was quantitated in cells treated with helenalin or/and Balifomycin A1, by trypsinizing and harvesting cells for FACS analysis. Approximately 80 % of cells treated with 2uM helenalin for 24 h were positive for AVO staining and these levels were completely abrogated by the addition of Bafilomycin A1 (Figure 5D).

### Inhibition of Atg12 and LC3-B expression reduces caspase cleavage and cell death induced by Helenalin

**Figure 6:**
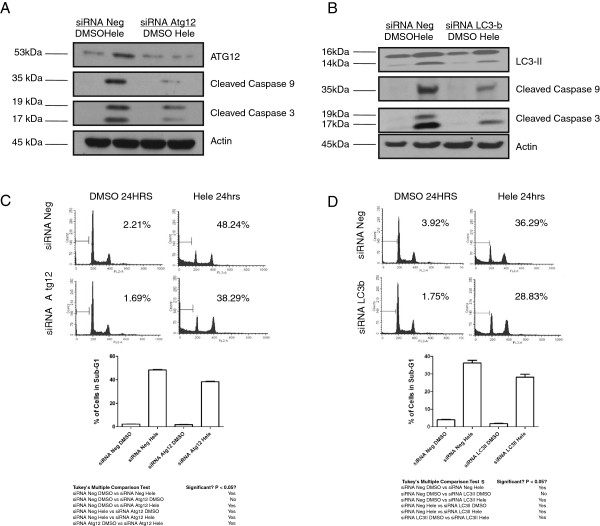
**Helenalin induced caspase cleavage and autophagy is dependent on Atg12 and LC3-B.** A2780 cells were transfected with either non-targeting siRNA (siRNA Neg) or Atg12 or LC3-B-specific siRNA for 24 h and then treated with either DMSO or helenalin for 24 h. Cells were harvested and (**A** and **B**) cell lysates were subjected to immunoblot analysis for Atg12, LC3-B, cleaved PARP, caspase 3 or 9 or (**C** and **D**) analyzed for cell cycle phase distribution by FACS. All experiments were performed in biological triplicates. Actin was used as the loading control. One way ANOVA was performed between control and treatment groups, and significant differences were observed between the groups as indicated in Figure 6C and D. Categories significant after multiple comparison are marked as “Yes”.(p-value <0.05; Tukey’s multiple comparison test).

To investigate the significance of Atg12 and LC3-B in cells undergoing helenalin induced autophagy, we depleted Atg12 and LC3-B in A2780 cells using siRNA-mediated knockdown. Post siRNA transfection and upon helenalin treatment, we observed a reduction of protein levels for both Atg12 and LC3-B when compared to cells treated with a non targeting control siRNA (Figure 6A and B). Intriguing, upon helenalin treatment, the protein levels of cleaved caspases were reduced in cells depleted of Atg12 and LC3-B as compared to cells treated with helenalin and transfected with a control non-targeting siRNA (Figure 6A and B). In addition, flow cytometry analysis performed in cells treated with Atg12 or LC3-B showed a decrease in sub-G1 levels when compared to cells treated with a non-targeting siRNA (Figure 6C and D). This result is consistent with previous findings where decrease in LC3-B was associated with reduced autophagy and cells treated with LC3-B or Beclin 1 siRNA inhibited caspase-3/8 activation [13]. In the context of helenalin induced cell death, this result implies that both Atg12 and LC3-B modulate caspase cleavage essential for autophagy.

### NF-κB p65 inhibition by Helenalin is essential for caspase cleavage and induction of autophagy

**Figure 7:**
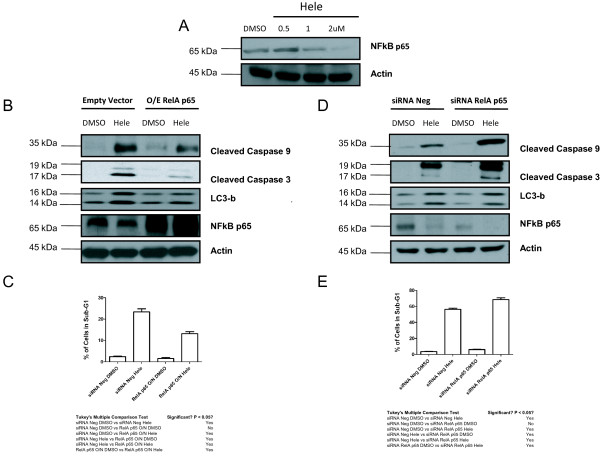
**Repression of NF-κB p65 expression by Helenalin is necessary for caspase cleavage and autophagy.** (**A**) A2750 cells were treated with increasing concentrations of helenalin for 24 h, following which cells were harvested and cell lysates prepared and subjected to immunoblot analysis for NF-κB. (**B**) A2780 cells were transfected with an empty vector or vector over-expressing RelA p65. 24 h post transfection, cells were treated with DMSO or 2uM helenalin for 24 h,and harvested for immunoblot analysis for cleaved caspase 3 and 9, *LC3-B* or NF-κB p65 or (**C**) trypsined and analyzed by FACS for precent of cells in sub-G1. One way ANOVA was performed for sub-G1 cells between control (DMSO) and treatment groups, and significant differences were observed between the groups as indicated in Figure 7C. Categories significant after multiple comparison are marked as “Yes”.(p-value <0.05; Tukey’s multiple comparison test). (**D**) A2780 cells were transfected either with a non-targeting siRNA (siRNA Neg) or siRNA targeting RelA p65. 24 h post transfection, cells were treated with DMSO or 2uM helenalin for 24 h and harvested for immunoblot analysis for cleaved caspase 3 and 9, LC3-B or NF-κB p65 or (**E**) trypsined and analyzed by FACS for percent of cells in sub-G1. One way ANOVA was performed for sub-G1 cells between control (DMSO) and treatment groups, and significant differences were observed between the groups as indicated in Figure 7E. Categories significant after multiple comparison are marked as “Yes”.(p-value <0.05; Tukey’s multiple comparison test). All experiments were performed in biological triplicates and actin was used as a loading control for immunoblot analysis.

To ascertain the mechanism by which helenalin induces Atg12 and LC3-B expression, we concerted our efforts in understanding the role of the transcription factor NF-κB p65 in helenalin induced autophagy. Previous reports have demonstrated helenalin’s role in anti-cancer and anti-inflammatory effects by inhibiting NF-κB and telomerase activity and impairing protein and DNA synthesis [6]. In addition, helenalin interacts with RelA to inhibit DNA binding to its cognate response elements and by inhibiting activation of the transcription factor NF-κB [7]. Blockade of NF-κB/p65 binding to DNA with helenalin correlated with induction of cell death in a dose-dependent manner [14]. Numerous reports have demonstrated NF-κB p65 to play a role in autophagy induced cell death [13,15], however the function in which helenalin participated in autophagy was unknown. Amalgamating this information, we decided to investigate NF-κB p65 for its role in helenalin induced autophagy cell death. As shown in Figure 7A, helenalin reduced the expression of NF-κB p65 in a dose dependent manner. Exogenous over-expression of NF-κB p65 reduced the levels of cleaved caspase 3, 9 and LC3-B in cells treated with helenalin (Figure 7B) with subsequent reduction of sub-G1 levels (Figure 7C), while siRNA mediated transcriptional knockdown of NF-κB p65 increased cleaved caspase 3 and 9 but not LC3-B after helenalin treatment (Figure 7D) with consequent increase in sub-G1 levels in cells (Figure 7E). No differences in LC3-B levels were observed after siRNA knockdown of NF-κB, since after helenalin treatment, NF-κB levels are reduced by the drug itself. This result was recapitulated in MCF-7 and RKO cells with analogous outcomes (data not shown).

## Conclusions

### Helenalin induces autophagy cell death *via* suppression of NF-κB p65

Pharmacologically active natural compounds such as those from marine and terrestrial plants and animals represent a promising resource for novel anticancer drugs. There are several prominent examples from the past proving the success of natural products and derivatives exhibiting anticancer activity. This includes the *Vinca* alkaloids from *Catharanthus roseus*, the terpene paclitaxel from *Taxus brevifolia*, the DNA topoisomerase I inhibitor camptothecin from *Camptotheca acuminata*, and the semisynthetic derivatives etoposide and teniposide of the lignan podophyllotoxin from *Podophyllum peltatum*. Natural products alone or synthetics developed based on knowledge gained from natural products account for about 70 % of anticancer therapeutics approved between 1980s and 2002 [16].

Herbal medicine has been applied in the clinic for thousands of years in Asian countries such as China, Japan and Korea [17]. However, because of its complicated chemical composition and lack of concrete evidence of its biological activity, herbal medicine is still not widely accepted by the Western medical community [18]. Unlike modern drugs in the form of a single active compound, herbal medicine is usually prepared from aqueous extracts of a few herbs and contains hundreds or even thousands of different compounds [19]. However, only a few compounds are responsible for the pharmacological effects [19]. Furthermore, the bioactive components are generally present at low level. Some components are useless or even toxic. Thus, systematic characterization of active chemicals in herbal medicinal preparations and their mechanisms of action are important for providing the rationale for their efficacy and for transforming herbal medicine practices into evidence-based medicine.

Helenalin, an extracted component of *Arnica Montana* and *Arnica chamissonis* is a sesquiterpene lactone with potent anti-inflammatory and antitumor activity [20]. The use of helenalin has been demonstrated to reduce the growth of *Staphylococcus aureus* and *Plasmodium falciparum*[21][22]*.*In addition, previous studies have implicated helenalin to selectively inhibit the transcription factor NF-κB [7] and human telomerase activity [5], suggesting an underlying molecular mechanism for its antitumor activity.

Our ensuing findings derived from experiments performed in cancer cells treated with helenalin consistently resulted in an increase in cell death *via* apoptosis and autophagy. The increased sensitivity to cell death when exposed to helenalin was associated with increased levels of caspase cleavage. Indeed, when caspase cleavage was blocked using a specific inhibitor, cell death was considerably reduced. Given that several anticancer agents exert their effects by triggering autophagy, we postulated whether helenalin’s action in triggering cell death was through the activation of autophagy. Treatment with helenalin resulted in an increase in defined autophagy markers, which when transcriptionally silenced using siRNA resulted in decreased cell death. Interestingly, transcriptionally silencing Atg12 and LC3-B, both essential for induction of autophagy cell death also resulted in a decrease of caspase activity. This result suggests that caspase activation is dependent on the expression of Atg12 and LC3-B. These observations are in agreement with previous studies where a decrease in LC3-B levels was associated with reduced autophagy and cells treated with LC3-B or Beclin 1 siRNA inhibited caspase-3/8 activation [13]. To further validate our findings, we performed Acridine Orange staining assays to measure acidic vesicular organelle (AVO) formation, a key indicator of autophagy initiation. AVO formation increased with increasing concentrations of helenalin and was suppressed with the use of bafilomycin A1, a highly potent and selective inhibitor of vacuolar H + −ATPases used in preventing the re-acidification of synaptic vesicles leading to the autophagy process [23-26].

We next examined whether helenalin’s mechanism of action was through the transcription factor NF-κB. Previous reports had revealed helenalin as a potent inhibitor of NF-κB [27] and that its binds with RelA disrupting its transcriptional activity [28]. In addition, NF-κB is a key regulator of several biological processes, including proliferation, differentiation, apoptosis and autophagy [29,30]. NF-κB has been demonstrated to play an essential role after heat shock treatment by modulating autophagy by a mechanism to increase cell survival, possibly through the elimination of irreversibly damaged proteins [31-33]. With this regard, we observe that upon helenalin treatment, the level of NF-κB p65 (RelA) was reduced. Reintroducing RelA exogenously *via* an over-expression construct we observed that caspase activation was reduced together with the levels of autophagy markers, resulting in decreased cell death. Conversely, transcriptionally silencing NF-κB p65 had the outcome of increasing caspase cleavage, autophagy markers and cell death. These results strongly advocate the reliance of NF-κB p65 for helenalin induced autophagy cell death. We speculate helenalin downregulates NF-κB p65 expression *via* ubiquitination-mediated degradation. Previous reports have shown that tumor necrosis factor-α (TNFα) polyubiquitinatates RelA at the lysine 195 residue which is critical for degradation of p65 [34]. The precise mechanism of p65 degradation needs to be further investigated.

In summary, we have shown that helenalin induces cell death *via* a mechanism involving the repression of NF-κB p65 expression resulting in an increase of autophagy markers and caspase activation. This provokes the clinically relevant question as to helenalin’s use as a therapeutic intervention in patients with aberrant activation of NF-κB. Clinically, acute myeloid leukemia (AML) is an aggressive cancer with median survival rates of 2 to 3 months, and inhibition of NF-κB is considered one of the therapeutic strategies for treatment [30,35-39]. Oncogenic addiction of activated NF-κB could be inhibited with the use of helenalin, and as such could favorably be used in a therapeutic setting to augment tumor sensitivity to conventional chemotherapeutic drugs. Further work is necessary before helenalin can be considered as a lead compound and a treatment strategy. Specificity, toxicology, pharmacokinetics and metabolism needs to be investigated and studied further before it is introduced into the market.

## Competing interests

The authors declare no competing financial interest and conflicts of interest with respect to the authorship and/or publication of this article.

## Authors’ contributions

N.K, P.Y.F, C.B.L and K.G.S carried out most of the experiments; C.B.L, M.S.H. and Z.Y. conceived and designed the project; J.L performed data analysis; M.S.H and Z.Y. interpreted the results and wrote the manuscript. All authors participated in data analysis. All authors read and edited the manuscript.

## Authors’ information

CBL and PYF are co-first authors.

## Supplementary Material

Additional file 1 ** Figure S1. Helenalin induces cell cycle arrest in G1 phase and modulates cell survival in a dose-dependent manner** ***.*** **(A)** MCF-7 cells were seeded onto 6-well plates and incubated for 24 h. Various concentrations of helenalin were added to the culture medium and incubated for an additional 24 h. Cells were then harvested and analyzed by flow cytometry. The cell cycle phase distribution was determined using CellQuest software.**(B)** Percent of viable MCF-7 cells after treatment with increasing concentrations of helenalin (serial dilution for drug concentration ranging from 10uM to 0.001uM) using the MTT assay. **(C)** RKO cells were seeded onto 6-well plates and incubated for 24 h. Various concentrations of helenalin were added to the culture medium and incubated for an additional 24 h. Cells were then harvested and analyzed by flow cytometry. The cell cycle phase distribution was determined using CellQuest software.**(D)** Percent of viable RKO cells after treatment with increasing concentrations of helenalin (serial dilution for drug concentration ranging from 10uM to 0.001uM) using the MTT assay. All experiments were performed in biological triplicates. Click here for file
